# Cleft Photo Mirror Combining Self-Retraction, Scaling, and Antifogging Features for Cleft Palate Documentation

**DOI:** 10.1097/PRS.0000000000012099

**Published:** 2025-03-17

**Authors:** Prasad Nalabothu, José Wittor de Macêdo Santos, Benito K. Benitez, Yoriko Lill, Sebastian Tapia Coron, Andreas A. Mueller

**Affiliations:** Basel, Switzerland; From the ^1^Department of Oral and Craniomaxillofacial Surgery, University Hospital Basel; 2 Facial and Cranial Anomalies Research Group, Department of Biomedical Engineering and Department of Clinical Research, University of Basel; 3 Department of Paediatrics Oral Health and Orthodontics, University Center for Dental Medicine UZB; 4 Pediatric Oral and Craniomaxillofacial Surgery, University Children’s Hospital Basel.

## Abstract

Documentation of patients with cleft lip and palate presents a significant challenge due to the necessity for consistent, high-quality imaging throughout the extended treatment period. The use of traditional intraoral mirrors typically necessitates an assisting person for retraction of the lips and cheeks, as well as mirror fog cleaning. This results in variable fields of view and requires extensive resources. Distance measurements are not possible on conventional photographs, limiting their use for objective treatment analysis. To address these 3 challenges at once, the authors developed a customized intraoral mirror incorporating self-retraction, a reference scale, and an antifogging option. The mirror design features lateral extensions for effective lip and cheek retraction and 2 small laser-engraved black triangles, spaced 2 cm apart at the end of the mirror, to measure the width of the cleft, enabling a single operator to capture clear images independently. The mirror is designed in 4 sizes to accommodate patients from newborn babies to adults. Clinical implementation demonstrated that the mirror allows the acquisition of high-quality standardized images without additional assistance, enhancing workflow efficiency and consistency in documentation. By overcoming the limitations of traditional mirrors, the authors’ mirror provides a practical and efficient solution for cleft lip and palate documentation and facilitates accurate follow-up of treatment progress beyond orthodontic and surgical evaluation.

Effective management of cleft lip and palate (CLP) necessitates long-term follow-up, with documentation using impressions, photographs, and videos.^1–3^ Such documentation is of utmost importance for evaluating surgical outcomes and growth. However, the quality of the registers is operator-dependent, as well as dependent on mirror quality and retraction. In response to this, we have developed a customized intraoral mirror designed specifically for photography in patients with cleft from birth to adulthood. These advancements allow a single operator to document the CLP efficiently, enhancing consistency.^4^

Our mirror was designed with 4 different prototype sizes, each mirror including lateral extensions for simultaneous self-retraction, ensuring that the lips and cheeks are effectively pulled aside to provide a clear view of the palate, alveolar ridges, and vestibulum (Fig. 1). The mirror sizes were developed based on interdisciplinary discussions among clinicians and previous literature indicating the intertuberosity distance on the cleft palate.^5–7^ These sizes ensure that the mirrors accommodate a wide range of anatomic and developmental profiles: size XS (35 mm) is tailored for newborns with cleft palate, size S (40 mm) is suitable for newborns with complete CLP, size M (45 mm) addresses infants up to approximately 1 to 2 years of age, and size L (50 mm) is designed for older children up to adulthood.

**Fig. 1.**
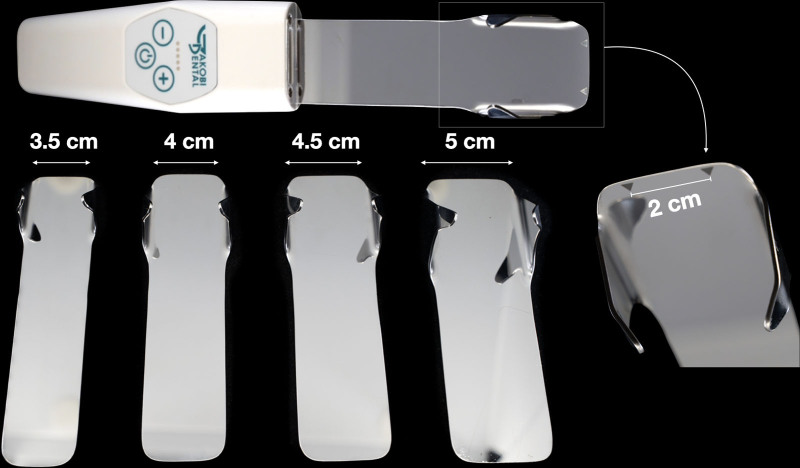
(*Above*) Support device with a built-in antifog fan and inserted mirror evidencing its arms for retraction. (*Right*) Close-up of the triangular markings used for standardized photographic measurements. (*Below*) Four different mirror sizes for adaptability on different patients.

The mirrors use medical-grade stainless steel and high-quality polished reflective surfaces to ensure durability and sharpness, allowing them to be warmed or sterilized (Jakobi Dental GmbH). Beyond the design, an important feature of our mirrors is compatibility with a support device that includes a small, battery-operated fan and light-emitting diode light source (Fig. 1). This device is strategically positioned at the mirror base insertion and allows a gentle airflow across the mirror’s surface, preventing fogging caused by the patient’s breathing or ventilation. The mirror and fan apparatus is lightweight and ergonomically designed, allowing for single-operator holding and positioning (Photo-Mirror-Demister; Jakobi Dental GmbH).

We implemented our intraoral mirror in a clinical setting to routinely document the palatal morphology of infants with CLP. The mirror can be used in an outpatient setting (Fig. 2) or in the operating room before or after surgery. Using our customized mirror, the clinicians could position the camera and capture high-quality images without assistance, representing a significant improvement over the standard mirrors typically used in similar procedures (Fig. 3, *left*). The mirror has engraved triangular markings with a 2-cm distance at the posterior end of the mirror for measurement of the transversal cleft width on the photographs (Fig. 3, *right*). Although plaster models are accurate, their workflow involves impressions that can compress the soft palate, causing minor inaccuracies. In contrast, the mirror method avoids soft-tissue displacement.

**Fig. 2.**
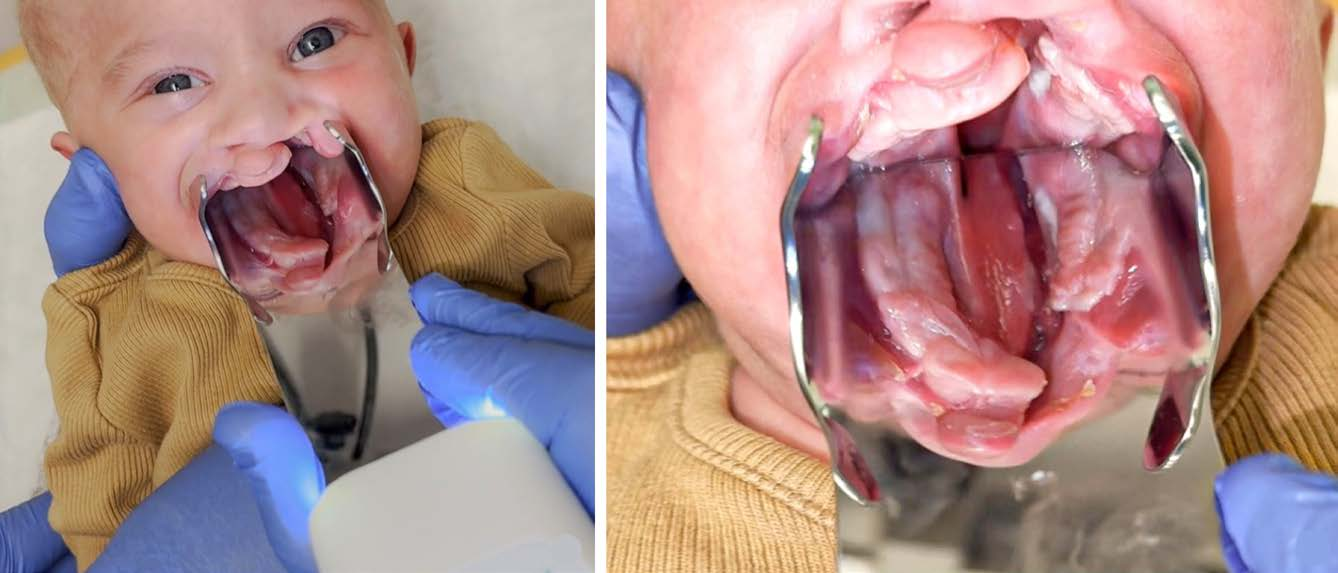
(*Left*) Positioning in outpatient settings for documentation by a single operator. (*Right*) Detailed anatomy of the hard palate cleft captured.

**Fig. 3.**
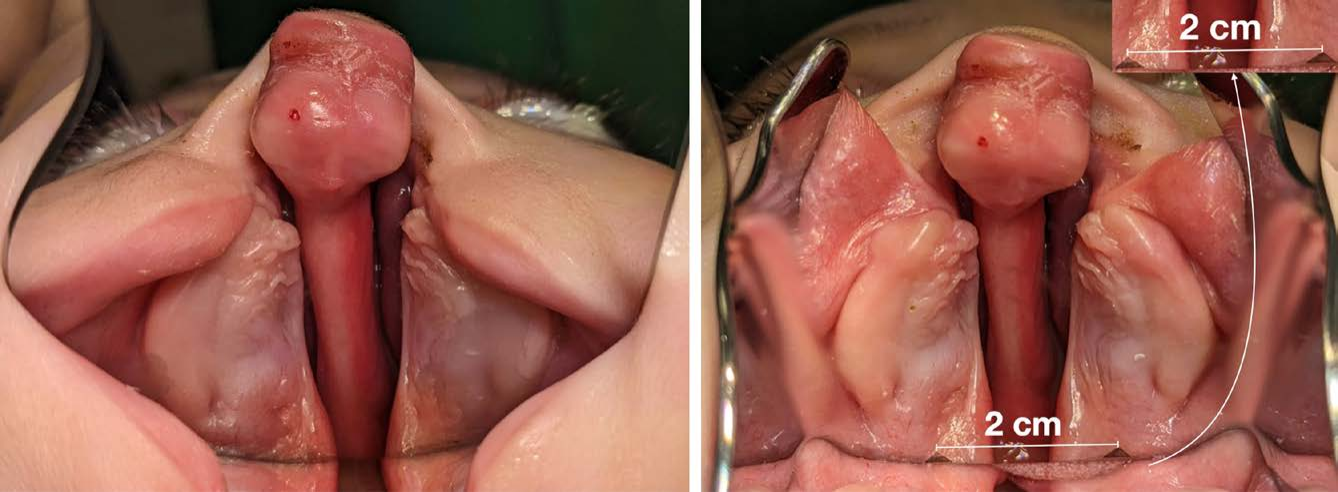
(*Left*) Presurgical documentation using a conventional mirror. (*Right*) Comparative intraoral picture using the novel self-retractable mirror featuring engraved triangles for width measurement.

The lateral extensions provide consistent retraction of the lips and cheeks while the mirror keeps the tongue retracted, and the antifogging fan ensures a clear reflective surface throughout the procedure. The images obtained are standardized and of high quality, with clear views of the cleft palate, aiding in accurate documentation and treatment planning.^8,9^ The supplemental videos highlight the mirror’s practical and user-friendly design in combination with a device. (**See Video 1 [online]**, which was recorded by a single operator in an outpatient setting, highlighting the practicality of the mirror and support assembly. **See Video 2 [online],** which shows an operating room demonstration of the mirror for presurgical photographic and video documentation.)

**Video 1.** This video was recorded by a single operator in an outpatient setting, highlighting the practicality of the mirror and support assembly.

**Video 2.** This video shows an operating room demonstration of the mirror for presurgical photographic and video documentation.

## DISCUSSION

Traditional intraoral mirrors present several challenges. First, an assistant is required to retract the lips and cheeks, which can result in inconsistencies and inefficiencies within clinical settings.^4,9,10^ Such reliance on additional personnel not only increases operational costs but also complicates scheduling and coordination within a busy health care environment. Second, it is not possible to measure the cleft width or how it has changed over time objectively. Third, photographs may be compromised by fogging due to patient breathing or ventilation, resulting in blurred images, creating a time-consuming task to capture a good image.^10^ The introduction of our self-retractable and scaled intraoral mirror represents a significant advancement in the field of cleft care documentation, addressing those previous long-standing challenges. (**See Video 3 [online]**, which shows the introduction of the mirror into an infant’s mouth, highlighting the retracting function.)

**Video 3.** This video shows the introduction of the mirror into an infant’s mouth, highlighting the retracting function.

Hospitals are progressively adopting electronic medical records, which facilitate the integration of diverse data types, including images.^11^ The use of smartphone cameras for clinical use facilitates streamlining of the multiprofessional workflow by capturing good images and integrating them into patient charts without expensive cameras.^12,13^

Our innovative intraoral mirror features scale markings, which are in close contact with the hard–soft palate junction. This design improves the accuracy of clinical assessments by allowing accurate measurements. Accurate measurement of cleft width is imperative, because cleft width is associated with the risk of fistula formation and hypernasality.^14,15^ By using this mirror, clinicians can objectively monitor changes over time, providing a valuable reference for surgical planning and enhancing outcomes.

Our mirror enables efficient single-operator documentation, minimizing the need for additional personnel or devices (although caregiver support may still be needed), simplifying the process in resource-limited and time-sensitive settings.^1^ The lateral extensions provide consistent retraction, ensuring a clear and unobstructed view of the palate. This feature is of particular importance for the capture of standardized photographs, which are necessary for the evaluation of the progress of CLP treatments.^1–6^ The availability of 4 different sizes allows use across a wide range of patient ages, from newborn to adult, thereby increasing the versatility and applicability of the device.

The connectivity of the mirror to a fan to prevent fogging is a strong asset. This maintains a clear reflective surface, conserving time during patient assessments. The ability of a single operator to manage both the retraction and photography is a game-changer in documenting CLP cases, optimizing clinic workflow and allowing for more consistent photographic documentation.^2–4,6^ This is particularly important for long-term patient monitoring, where consistency in image quality and perspective is essential for tracking treatment progress and outcomes.^1–6^ This innovation falls within the growing trend of digitization in health care, integrating seamlessly with electronic medical records and smartphone-based imaging.

## CONCLUSIONS

Our customized cleft palate intraoral mirrors with self-retraction, scaling, and antifogging features present an improvement in the documentation of patients with CLP, overcoming the key challenges of traditional methods, and offering a practical, efficient, and high-quality solution for clinicians, orthodontists, and surgeons to record intraoral cleft palate photographs.

## DISCLOSURE

The authors have no conflicts of interest to disclose.

## PATIENT CONSENT

Parents or guardians provided written informed consent for use of patients’ images.

## ACKNOWLEDGMENTS

This innovation was supported by the Basel Research Centre for Child Health (BRCCH), supporting the Research Consortium for Pediatric Digital Health with a Multi-Investigator Project “Burden-Reduced Cleft Lip and Palate Care and Healing,” led by Dr. Mueller, principal investigator. The funding sources were not involved in the study design; the collection, analysis, or interpretation of data; the preparation of the manuscript; or the decision to submit the manuscript for publication. A European patent application was filed for the mirror device before publication of this article. The authors thank Jakobi Dental GmbH for providing the mirrors, Maren Roche for coordination, and the patients and their parents for participation.
